# Endovascular Thrombectomy for Ischemic Stroke Increases Disability-Free Survival, Quality of Life, and Life Expectancy and Reduces Cost

**DOI:** 10.3389/fneur.2017.00657

**Published:** 2017-12-14

**Authors:** Bruce C. V. Campbell, Peter J. Mitchell, Leonid Churilov, Mahsa Keshtkaran, Keun-Sik Hong, Timothy J. Kleinig, Helen M. Dewey, Nawaf Yassi, Bernard Yan, Richard J. Dowling, Mark W. Parsons, Teddy Y. Wu, Mark Brooks, Marion A. Simpson, Ferdinand Miteff, Christopher R. Levi, Martin Krause, Timothy J. Harrington, Kenneth C. Faulder, Brendan S. Steinfort, Timothy Ang, Rebecca Scroop, P. Alan Barber, Ben McGuinness, Tissa Wijeratne, Thanh G. Phan, Winston Chong, Ronil V. Chandra, Christopher F. Bladin, Henry Rice, Laetitia de Villiers, Henry Ma, Patricia M. Desmond, Atte Meretoja, Dominique A. Cadilhac, Geoffrey A. Donnan, Stephen M. Davis, on behalf of the EXTEND-IA Investigators, Stephen M Davis

**Affiliations:** ^1^Department of Medicine and Neurology, Melbourne Brain Centre at the Royal Melbourne Hospital, University of Melbourne, Parkville, VIC, Australia; ^2^Department of Radiology, The Royal Melbourne Hospital, University of Melbourne, Parkville, VIC, Australia; ^3^The Florey Institute of Neuroscience and Mental Health, University of Melbourne, Parkville, VIC, Australia; ^4^Department of Neurology, Ilsan Paik Hospital, Inje University, Gyeonggi-do, South Korea; ^5^Royal Adelaide Hospital, Adelaide, SA, Australia; ^6^Department of Neurosciences, Eastern Health and Eastern Health Clinical School, Monash University, Clayton, VIC, Australia; ^7^Priority Research Centre for Brain and Mental Health Research, John Hunter Hospital, University of Newcastle, Newcastle, NSW, Australia; ^8^Austin Health, Heidelberg, VIC, Australia; ^9^Department of Radiology, Royal North Shore Hospital, St Leonards, NSW, Australia; ^10^Department of Neurology, Royal North Shore Hospital, Kolling Institute, University of Sydney, St Leonards, NSW, Australia; ^11^Centre for Brain Research, University of Auckland, Auckland City Hospital, Auckland, New Zealand; ^12^Auckland City Hospital, Auckland, New Zealand; ^13^Western Hospital, Footscray, VIC, Australia; ^14^Monash Medical Centre, Monash University, Clayton, VIC, Australia; ^15^Gold Coast University Hospital, Southport, QLD, Australia; ^16^Department of Neurology, Helsinki University Hospital, Helsinki, Finland; ^17^Stroke and Ageing Research, Department of Medicine, School of Clinical Sciences at Monash Health, Monash University, Clayton, VIC, Australia

**Keywords:** ischemic stroke, thrombolysis, endovascular therapy, mechanical thrombectomy, intraarterial therapy, Solitaire stent retriever device, CT perfusion, randomized trial

## Abstract

**Background:**

Endovascular thrombectomy improves functional outcome in large vessel occlusion ischemic stroke. We examined disability, quality of life, survival and acute care costs in the EXTEND-IA trial, which used CT-perfusion imaging selection.

**Methods:**

Large vessel ischemic stroke patients with favorable CT-perfusion were randomized to endovascular thrombectomy after alteplase versus alteplase-only. Clinical outcome was prospectively measured using 90-day modified Rankin scale (mRS). Individual patient expected survival and net difference in Disability/Quality-adjusted life years (DALY/QALY) up to 15 years from stroke were modeled using age, sex, 90-day mRS, and utility scores. Level of care within the first 90 days was prospectively measured and used to estimate procedure and inpatient care costs (US$ reference year 2014).

**Results:**

There were 70 patients, 35 in each arm, mean age 69, median NIHSS 15 (IQR 12–19). The median (IQR) disability-weighted utility score at 90 days was 0.65 (0.00–0.91) in the alteplase-only versus 0.91 (0.65–1.00) in the endovascular group (*p* = 0.005). Modeled life expectancy was greater in the endovascular versus alteplase-only group (median 15.6 versus 11.2 years, *p* = 0.02). The endovascular thrombectomy group had fewer simulated DALYs lost over 15 years [median (IQR) 5.5 (3.2–8.7) versus 8.9 (4.7–13.8), *p* = 0.02] and more QALY gained [median (IQR) 9.3 (4.2–13.1) versus 4.9 (0.3–8.5), *p* = 0.03]. Endovascular patients spent less time in hospital [median (IQR) 5 (3–11) days versus 8 (5–14) days, *p* = 0.04] and rehabilitation [median (IQR) 0 (0–28) versus 27 (0–65) days, *p* = 0.03]. The estimated inpatient costs in the first 90 days were less in the thrombectomy group (average US$15,689 versus US$30,569, *p* = 0.008) offsetting the costs of interhospital transport and the thrombectomy procedure (average US$10,515). The average saving per patient treated with thrombectomy was US$4,365.

**Conclusion:**

Thrombectomy patients with large vessel occlusion and salvageable tissue on CT-perfusion had reduced length of stay and overall costs to 90 days. There was evidence of clinically relevant improvement in long-term survival and quality of life.

**Clinical Trial Registration:**

http://www.ClinicalTrials.gov NCT01492725 (registered 20/11/2011).

## Introduction

Multiple randomized trials have demonstrated improved outcomes with endovascular stent thrombectomy compared to standard care in patients with ischemic stroke due to large vessel occlusion within 6 h of onset (1–6). The effect size was substantial with individual patient data meta-analysis indicating a number needed to treat (NNT) to improve disability by at least one level on the modified Rankin Scale (mRS) of 2.6, and an NNT to achieve an extra disability-free outcome of 5 (7).

Disability-adjusted life years (DALY) and quality-adjusted life years (QALY) are summative population outcome metrics used to describe the impact of stroke recovery and weight the patient’s remaining years of life either by the degree of loss of function (DALY) or quality of life (QALY) (8). These metrics are often used to describe the burden of disease or provide the benefit (effectiveness) component used in cost-effectiveness evaluations.

Since 2015, several formal cost-effectiveness analyses have been published on endovascular stent thrombectomy. A composite of functional outcomes from the MR CLEAN trial and US administrative costing data were used in one analysis that found endovascular thrombectomy was cost-effective with an average US$14,137 per QALY gained and simulations providing evidence of a 97.6% likelihood of being <US$50,000 per QALY over the patients lifetime using a social perspective (9). Similarly, in modeling based on the United Kingdom health system and data from the initial five positive trials, endovascular thrombectomy cost an average $11,651 (£7,061) per QALY gained over the patient’s lifetime with 100% likelihood of being <US$33,000 per QALY using the perspective of the UK National Health Service and Personal Social Services (10). Using similar data from the five trials but from a Swedish health care payer perspective, a small cost saving over the patient’s lifetime and improvement by approximately one QALY was demonstrated (11). A study based on the SWIFT PRIME trial, which had one of the larger treatment effects among the trials, a major cost savings over the patient’s lifetime of $23,203 per patient using the perspective of the US health care system were reported (12). The THRACE trial had a smaller effect size and utilized a short 1-year time horizon but demonstrated an incremental cost per one QALY gained of $14,881 (€13,423) using the perspective of the French national health system (13).

A unique feature of the Australian-led EXTEND-IA randomized controlled trial (2) was that all patients were selected on the basis of CT perfusion imaging evidence of salvageable brain tissue. Endovascular thrombectomy significantly improved functional outcome (day 90 mRS) with 71% achieving functional independence versus 40% in the alteplase-only group. We aimed to estimate the potential impact on resource costs and disability-adjusted survival benefits of stent thrombectomy compared to intravenous thrombolysis alone in patients selected using perfusion imaging.

## Materials and Methods

In the EXTEND-IA trial, 70 patients were randomized from 10 hospitals in Australia and New Zealand between August 2012 and October 2014. The detailed trial protocol (14) and results (2) have been published. Briefly, patients were eligible if they were receiving intravenous alteplase within 4.5 h of stroke onset and had large vessel intracranial occlusion (internal carotid or middle cerebral M1 or M2 arteries) and evidence of salvageable brain tissue using CT perfusion with irreversibly injured ischemic core <70mL (using automated RAPID software, Stanford University (15, 16)). Eligibility criteria did not specify an upper age limit or any clinical severity limits using the National Institutes of Health Stroke Scale (NIHSS) score. Patients were randomized 1:1 to endovascular thrombectomy with the Solitaire FR device after alteplase versus alteplase alone. Functional outcome was assessed using the mRS at 90 days. This trial was carried out in accordance with the recommendations of the National Statement on Ethical Conduct in Human Research 2007 of the National Health and Medical Research Council of Australia with written informed consent from all participants or their legal representative in accordance with the Declaration of Helsinki. The protocol was approved by the Melbourne Health Human Research Ethics Committee.

Average utility values for disability derived from both patient- and clinician-based studies have been recently published for each level of the mRS: utility was scored 1.0 for mRS = 0; 0.91 for mRS = 1; 0.76 for mRS = 2; 0.65 for mRS = 3; 0.33 for mRS = 4; 0 for mRS = 5; and 0 for mRS = 6 (17). These were applied to the mRS at 90 days to compare the level of disability between groups. Similarly, QALY benefits were calculated using published quality of life utility values for each level of the mRS: 0.8 for mRS = 0; 0.8 for mRS = 1; 0.65 for mRS = 2; 0.50 for mRS = 3; 0.35 for mRS = 4; 0.20 for mRS = 5; and 0 for mRS = 6 (18). An association between mRS score at 90 days and subsequent mortality rate has been previously demonstrated (19). In this study, we extrapolated the expected survival for each patient in EXTEND-IA based on the published hazard ratios and age- and sex-specific life expectancy drawn from Australian Bureau of Statistics life expectancy data for 2013–2015 (20).

Detailed data on length of stay in the acute stroke unit, inpatient fast and slow (“geriatric”) stream rehabilitation, nursing home and palliative care were prospectively collected for each patient, including home time as the total number of days spent home within the first 90 days (21). Costs per day for each level of care in the Australian health care system in 2014 (Table 1) were supplied by the Royal Melbourne Hospital Finance Department (22) and converted to US dollars using the purchasing power parity of 68.54 cents per Australian dollar for 2014 (23).

**Table 1:** Admission costs per day.

Setting	Cost per day (US dollars)
Acute stroke unit	$1,053
Intensive care unit	$133 per hour
Fast stream inpatient rehabilitation	$533
Slow stream geriatric rehabilitation	$434
Nursing home care/skilled nursing facility	$175

*Data provided by the Royal Melbourne Hospital Finance Department based on 2014 costings (22). Conversion from Australian to US dollars using 2014 OECD purchasing price power parity (0.6854) (23)*.

Costs of the endovascular procedure were calculated including consumables and staffing as detailed in Table 2. The average staffing cost allowed for one neurointerventionist, one neurointerventional fellow, one anesthesiologist, one radiographer, and three nurses for 2 h with an extra hour to cover travel time for after hours cases, and loss of the radiographer and two nurses for 4 h the following day for cases after midnight. We allowed for 50% of cases in-hours and 50% out of hours (including 10% after midnight) based on the rates at our center which operated 24 h a day. This more realistically represented current clinical practice, in contrast to most other centers participating in EXTEND-IA that recruited trial participants only during the day.

**Table 2:** Average cost per patient by treatment group (US dollars).

Resource use and costs	Alteplase only	Alteplase + endovascular
Alteplase per patient	$3,167	$3,167
Interhospital transfer (allowing 75% transferred, metropolitan region)^a^	n/a	$573
Endovascular consumables^b^	n/a	$7,327
Endovascular staffing^c^	n/a	$2,615
Inpatient care costs $ (95% CI)	$30,569 ($21,575–$39,564)	$15,689 ($10,774–$20,605)
**TOTAL**	**$33,736**	**$29,371**
*Worst case sensitivity analysis*^d^	*$24,742*	*$34,287*

*Conversion from Australian to United States (US) dollars using 2014 OECD purchasing power parity (0.6854) (23)*.

*^a^75% of cases transferred based on Royal Melbourne Hospital experience, average ambulance cost from Ambulance Victoria within metropolitan area US$764, transfers from rural areas are more expensive*.

*^b^8Fr sheath, microcatheter, guidewires, balloon guide catheter, average 1.1 Solitaire devices per patient, groin closure device, and miscellaneous consumables*.

*^c^One neurointerventionist, one neurointerventional fellow, one anesthesiologist, one radiographer, and three nurses for 2 h with an extra hour to cover travel time for after hours cases and loss of the radiographer and two nurses for 4 h the following day for cases after midnight. We allowed for 50% of cases in-hours and 50% out of hours (including 10% after midnight) based on the rates at our center*.

*^d^Worst-case sensitivity analysis using the lower 95% confidence interval for the mean cost in alteplase-only patients and the upper 95% confidence interval for the mean cost in endovascular patients*.

### Statistical Analysis

Expected survival in the endovascular and alteplase-only groups was compared in a Cox regression model censored at 15 years post-stroke. Survival estimates were combined with utility values (assuming no change in mRS between 3 months and death) to evaluate lifetime DALY gained with endovascular versus alteplase-only treatment, and the incremental cost-effectiveness ratio (ICER) per DALY.

Disability-adjusted life years benefit was calculated with and without age-weighting and discounting. Age-weighting reflects a view that years lost beyond the age of 63 are of progressively less value than those lost during working life. Discounting reduces the value of disability-free life by 3% per annum. Currently, the World Health Organization (WHO) does not apply age-weighting or discounting for DALY calculations (24). QALY benefit is generally not age-weighted but can be discounted. We have primarily reported results without age-weighting or discounting but provided the alternative combinations, according to standard methodology (25), for sensitivity analysis.

Length of stay data were compared between treatment groups (Wilcoxon test). The cost of inpatient and nursing home care within the first 90 days was calculated for each patient based on treatment and bed costs. For sensitivity analysis, a “worst case” cost difference was calculated using the upper 95% confidence interval for the mean endovascular cost and the lower 95% confidence interval for mean alteplase-only cost.

## Results

There were 70 patients randomized (35 in each arm) with mean age of 69 (SD 12) years, median NIHSS score 15 (IQR 12–19), and 49% male sex. Detailed patient characteristics were published previously and there were no statistically significant imbalances (2). At the time of stroke, 24/70 (34%) were aged 65 years or younger and in paid employment.

**Figure 1:**
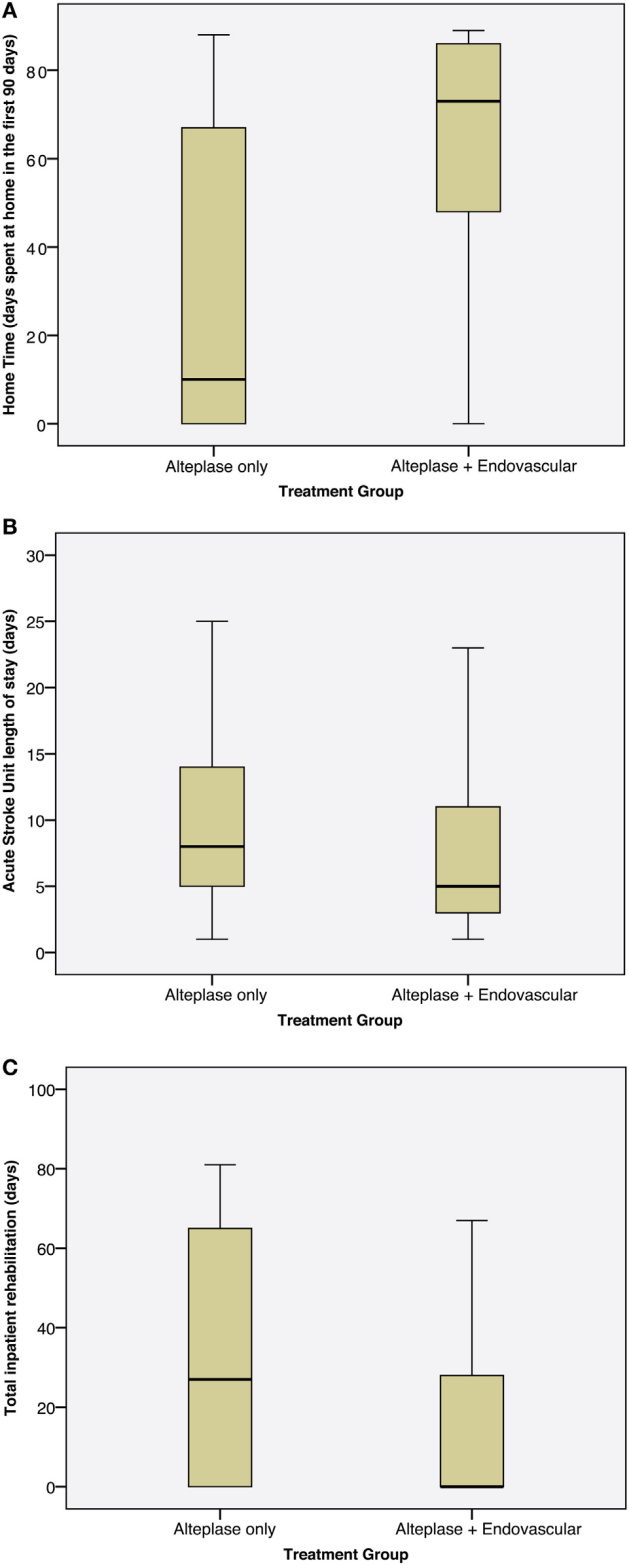
Boxplots showing length of stay by treatment group. **(A)** Home time (number of days spent at home in the first 90 days post-stroke), **(B)** length of stay in the acute stroke unit, **(C)** length of stay in inpatient rehabilitation.

Home time (days spent at home in the first 90 days) was increased from median (IQR) 15 (0–69) in the control group to 73 (47–86) days in the endovascular group, *p* = 0.001. Length of stay in the acute stroke unit was reduced from median (IQR) 8 (5–14) days in the control group to 5 (3–11) days in the endovascular group (mean 12 versus 8 days), *p* = 0.04 with no increase in intensive care utilization [median (IQR) 0 (0–24) hours in control versus 0 (0–4) hours in the endovascular group (mean 11 versus 9 h), *p* = 0.51]. Rehabilitation length of stay in survivors was reduced from median (IQR) 27 (0–65) days in the control group to 0 (0–28) days in the endovascular group (mean 33 versus 14 days), *p* = 0.03 with 25% of endovascular patients discharged directly home from the acute stroke unit (Figure 1).

**Figure 2:**
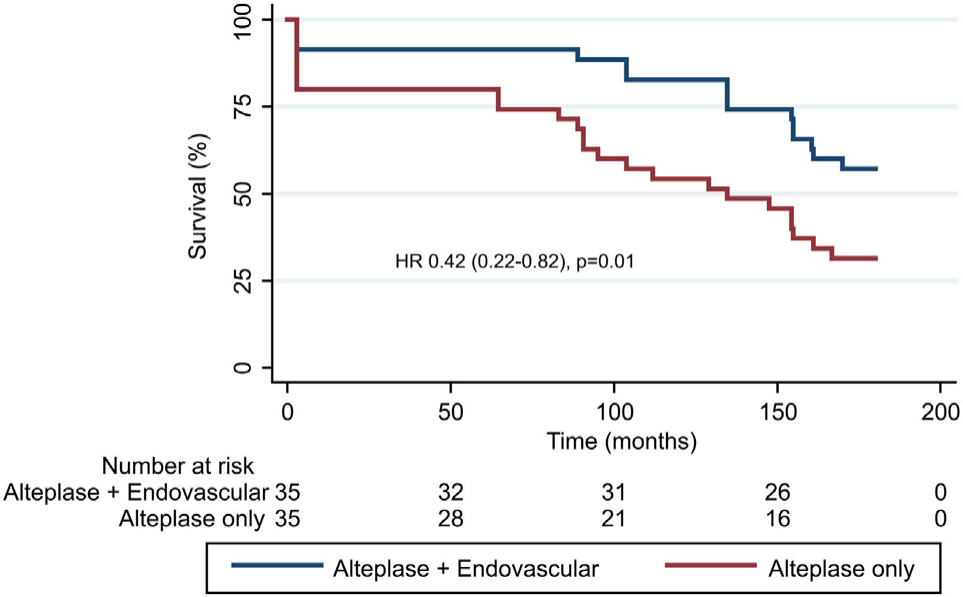
Projected survival modeled over 15 years (using extrapolation from 3 months modified Rankin Scale). The adjusted hazard ratio from Cox regression model was 0.42 (0.22–0.82), *p* = 0.01.

Based on the published utility weighting for mRS, the median utility score at 90 days was 0.65 (IQR 0–0.91) in the control versus 0.91 (IQR 0.65–1) in the endovascular group (unadjusted *p* = 0.005, adjusted for age and baseline NIHSS *p* = 0.02), an increase of approximately one quarter in the total utility range of 0–1. Based on the 90-day mRS distributions observed, a greater median life expectancy in the endovascular versus control group (15.6 versus 11.2 years, *p* = 0.02) was found using long-term simulation modeling. In a Cox regression model censored at 15 years post-treatment and adjusted for age and baseline NIHSS, endovascular treatment was associated with reduced risk of death (HR 0.42, CI_95_ 0.22–0.82, *p* = 0.01, Figure 2).

Applying utility scores, median DALYs lost in the endovascular group over the projected lifespan were 5.5 (IQR 3.2–8.7) compared to 8.9 (IQR 4.7–13.8) in the control group (*p* = 0.02, adjusted for baseline age and NIHSS) without age-weighting or discounting (age-weighted and discounted data in Table 3).

**Table 3:** Disability-adjusted life years (DALY) lost and Quality-adjusted life years (QALY) gained in endovascular versus alteplase-only groups calculated with and without age-weighting and discounting.

	Median years (IQR) endovascular versus alteplase-only	*p*-Value		Worst-case ICER (US dollars)
		Unadjusted	Adjusted for age and baseline NIHSS	
**DALY lost**				
Not discounted not age-weighted	5.5 (3.2–8.7) versus 8.9 (4.7–13.8) (mean difference 2.72)	0.04	0.02	$3,509
Discounted not age-weighted	3.2 (1.9–6.2) versus 6.3 (2.9–10.5) (mean difference 2.34)	0.02	0.02	$4,079
Not discounted age-weighted	2.3 (1.6–5.0) versus 4.1 (2.2–7.5) (mean difference 1.45)	0.07	0.02	$6,583
Discounted age-weighted	2.0 (0.94–3.6) versus 3.5 (1.6–5.8) (mean difference 1.27)	0.03	0.02	$7,516

**QALY gained**				
Not discounted	9.3 (4.2–13.1) versus 4.9 (0.3–8.3) (mean difference 3.29)	0.01	0.03	$2,901
Discounted	7.5 (2.7–10.2) versus 4.0 (0.3–6.9) (mean difference 2.50)	0.01	0.03	$3,818

*Worst-case incremental cost-effectiveness ratios (ICER) based on upper 95% confidence interval for endovascular cost and lower 95% confidence interval for alteplase-only cost*.

Applying quality of life weighting, median QALYs gained in the endovascular group over the projected lifespan were 9.3 (IQR 4.2–13.1) compared to 4.9 (IQR 0.3–8.5) in control group (*p* = 0.03, adjusted for baseline age and NIHSS) without discounting (discounted data in Table 3).

The average cost of inpatient care in the first 90 days was estimated to be US$14,880 less for endovascular compared to control patients (*p* = 0.008), with the average per patient cost US$4,365 less in the endovascular group after accounting for inter-hospital transfers and thrombectomy procedural costs (Table 2). Therefore, endovascular therapy was determined to be cost-saving (or “dominant” in health-economic terms) in this context. Ongoing nursing care (US$70,000 p.a.) was required for 0/35 endovascular and 5/35 (14%) alteplase-only patients (*p* = 0.054).

In sensitivity analyses, the “worst case” additional cost of endovascular thrombectomy was US$9,545 which generated an ICER of US$3,509 to US$7,516 per DALY lost depending on whether age-weighting and discounting were applied; and US$2,901 to $US3,818 per QALY gained depending on whether discounting was applied (Table 3).

## Discussion

In patients with ischemic stroke due to proximal cerebral arterial occlusion who had salvageable tissue on CT-perfusion imaging, endovascular thrombectomy combined with intravenous alteplase reduced disability and length of stay within the first 90 days compared with alteplase alone. Overall, healthcare utilization was reduced leading to estimated cost savings by 90 days. Based on simulation modeling of 90-day mRS scores, Australian life table data, and expected mortality rates by mRS category, a sustained and statistically significant mortality benefit up to 15 years post-treatment was found with associated benefits in DALYs lost and QALYs gained. On average, endovascular thrombectomy saved approximately two DALYs over the patient’s lifetime and US$4,365 per patient within the first 90 days. The worst case ICER of US$7,516 per DALY or US$3,818 per QALY is well within the $50,000 “willingness to pay” criterion often applied. This sensitivity analysis using the most pessimistic endovascular and optimistic alteplase-only costing suggests robust cost-effectiveness, as supported by other studies that demonstrated cost-effectiveness and, in many cases, cost savings with endovascular thrombectomy (9–13). Given the substantially greater level of disability in the alteplase-only group, the costs of care beyond 90 days, which were not included in this analysis, would be expected to remain greater than in the endovascular group.

EXTEND-IA had the largest absolute benefit of endovascular therapy of the published trials, likely due to rapid and effective revascularization combined with perfusion imaging selection to exclude patients with large areas of irreversibly injured brain. However, the relative risk of independent outcome was very similar among all the stent retriever trials (26). Therefore, although costs of care for the endovascular group in other trials may be greater, there would also likely be an increase in the costs of care for the control group.

Strengths of our study include the prospective design nested within a randomized controlled trial that utilized a standardized selection and treatment protocol. Limitations include the relatively small sample size introducing imprecision to our estimates. However, EXTEND-IA prospectively collected length of stay data and the effect size was sufficient to provide statistically robust results. Costs of care were estimated based on utilization of bed-days or ICU hours with fixed costs for these from one large Australian hospital that contributed the majority of cases. In reality, individual patients with similar number of bed days may require different intensity of management with medications, imaging, pathology, nursing and allied health interventions which, depending on the local payment system, may lead to variation in true patient costs. Our quality of life weights were based on published averages for mRS categories that could vary between cultures. Our long-term life expectancy data were based on extrapolation and modeling. We did not follow-up patients beyond 90 days but the long-term stability of the endovascular thrombectomy treatment effect has been established in other trials in analyses that concluded that 90-day follow-up was sufficient to provide a reliable estimate of disability status (27, 28). EXTEND-IA (and all the randomized trials of thrombectomy) excluded patients with pre-morbid disability. Our results would therefore not apply to treatment of patients with pre-existing disability, as may occur in clinical practice. Importantly, elderly patients (who have less potential QALY gain) were not excluded from EXTEND-IA which increases the generalizability of our results. As with any cost-utility analyses, generalizability can be questioned when health systems differ in the setting for acute stroke management, discharge practices, and costs. For example, US patients receive neurocritical care more often than patients in the Australian system and are discharged to rehabilitation facilities at an earlier stage (29). However, the magnitude of difference in inpatient care costs suggests that this finding is likely to be applicable to other health systems.

We conclude that endovascular thrombectomy in patients with favorable CT perfusion imaging has a major impact in reducing disability and improving quality of life after large vessel ischemic stroke. These improvements may lead to substantial cost savings.

## Ethics Statement

This study was carried out in accordance with the recommendations of the National Statement on Ethical Conduct in Human Research 2007 of the National Health and Medical Research Council of Australia with written informed consent from all participants or their legal representative in accordance with the Declaration of Helsinki. The protocol was approved by the Melbourne Health Human Research Ethics Committee.

## Author Contributions

BC was co-principal investigator and medical co-ordinator of the EXTEND-IA trial. He obtained funding and managed the trial operations, analyzed results and drafted the manuscript. PM was co-principal investigator and chair of neurointervention for the EXTEND-IA trial. He edited the manuscript. LC and MK performed statistical analyses and edited the manuscript. K-SH assisted with statistical analyses and edited the manuscript. AM and DC provided advice on study design and analysis and edited the manuscript. SD and GD were co-chairs of the EXTEND-IA steering committee and edited the manuscript. All other authors collected data and edited the manuscript.

## Conflict of Interest Statement

A/Prof Meretoja has consulted for and received honoraria for talks from Boehringer Ingelheim, manufacturer of alteplase, and Stryker, manufacturer of endovascular devices for ischemic stroke. A/Prof Cadilhac has received unrestricted education grants for work unrelated to this study from Boehringer Ingelheim and Medtronic. All other authors declare that the research was conducted in the absence of any commercial or financial relationships that could be construed as a potential conflict of interest.
