# Supplementary material for: Endovascular Thrombectomy for Ischemic Stroke Increases Disability-Free Survival, Quality of Life, and Life Expectancy and Reduces Cost
Source: Front Neurol. 2017 Dec 14;8:657. doi: 10.3389/fneur.2017.00657 (PMC5735082; doi:10.3389/fneur.2017.00657)
Supplement: Supplementary file 1 [file data_sheet_1.docx]

| **First names** | **Last names** |
| --- | --- |
| Stephen M | Davis |
| Geoffrey A | Donnan |
| Bruce CV | Campbell |
| Peter J | Mitchell |
| Leonid | Churilov |
| Bernard | Yan |
| Richard | Dowling |
| Nawaf | Yassi |
| Thomas J | Oxley |
| Teddy Y | Wu |
| Gabriel | Silver |
| Amy | McDonald |
| Rachael | McCoy |
| Timothy J | Kleinig |
| Rebecca | Scroop |
| Helen M | Dewey |
| Marion | Simpson |
| Mark | Brooks |
| Bronwyn | Coulton |
| Martin | Krause |
| Timothy J | Harrington |
| Brendan | Steinfort |
| Kenneth | Faulder |
| Miriam | Priglinger |
| Susan | Day |
| Thanh | Phan |
| Winston | Chong |
| Michael | Holt |
| Ronil V | Chandra |
| Henry | Ma |
| Dennis | Young |
| Kitty | Wong |
| Tissa | Wijeratne |
| Hans | Tu |
| Elizabeth | Mackay |
| Sherisse | Celestino |
| Christopher F | Bladin |
| Poh Sien | Loh |
| Amanda | Gilligan |
| Zofia | Ross |
| Skye | Coote |
| Tanya | Frost |
| Mark W | Parsons |
| Ferdinand | Miteff |
| Christopher R | Levi |
| Timothy | Ang |
| Neil | Spratt |
| Lara | Kaauwai |
| Monica | Badve |
| Henry | Rice |
| Laetitia | de Villiers |
| P. Alan | Barber |
| Ben | McGuinness |
| Ayton | Hope |
| Maurice | Moriarty |
| Patricia | Bennett |
| Andrew | Wong |
| Alan | Coulthard |
| Andrew | Lee |
| Jim | Jannes |
| Deborah | Field |
| Gagan | Sharma |
| Simon | Salinas |
| Elise | Cowley |
| Barry | Snow |
| John | Kolbe |
| Richard | Stark |
| John | King |
| Richard | Macdonnell |
| John | Attia |
| Cate | D’Este |
